# Effects of Aroma Massage on Home Blood Pressure, Ambulatory Blood Pressure, and Sleep Quality in Middle-Aged Women with Hypertension

**DOI:** 10.1155/2013/403251

**Published:** 2013-01-30

**Authors:** Myeong-Sook Ju, Sahng Lee, Ikyul Bae, Myung-Haeng Hur, Kayeon Seong, Myeong Soo Lee

**Affiliations:** ^1^Eulji University Hospital, Daejeon 302-799, Republic of Korea; ^2^Smart Hospital, Daejeon 302-859, Republic of Korea; ^3^Kunsan College of Nursing, Gunsan 573-719, Republic of Korea; ^4^College of Nursing, Eulji University, 143-5 Yongdudong, Jung-gu, Daejeon 302-832, Republic of Korea; ^5^Medical Research Division, Korea Institute of Oriental Medicine, Daejeon 305-811, Republic of Korea

## Abstract

The purpose of this study was to evaluate the effects of aroma massage applied to middle-aged women with hypertension. The research study had a nonequivalent control group, nonsynchronized design to investigate the effect on home blood pressure (BP), ambulatory BP, and sleep. The hypertensive patients were allocated into the aroma massage group (*n* = 28), the placebo group (*n* = 28), and the no-treatment control group (*n* = 27). To evaluate the effects of aroma massage, the experimental group received a massage with essential oils prescribed by an aromatherapist once a week and body cream once a day. The placebo group received a massage using artificial fragrance oil once a week and body cream once a day. BP, pulse rate, sleep conditions, and 24-hour ambulatory BP were monitored before and after the experiment. There was a significant difference in home systolic blood pressure (SBP) (*F* = 6.71, *P* = 0.002) between groups after intervention. There was also a significant difference in SBP (*F* = 13.34, *P* = 0.001) and diastolic blood pressure (DBP) (*F* = 8.46, *P* = 0.005) in the laboratory between aroma massage and placebo groups. In sleep quality, there was a significant difference between groups (*F* = 6.75, *P* = 0.002). In conclusion, aroma massage may help improve patient quality of life and maintain health as a nursing intervention in daily life.

## 1. Introduction

A chronic disease refers to a disease state progressing over a long period of time, and chronic diseases are responsible for 63% of overall deaths worldwide [1]. Of the chronic diseases in South Korea, the prevalence of hypertension in people over the age of 30 increased from 24.6% in 2007 to 26.9% in 2008 [2]. In particular, women show an increasing prevalence with age (4.3% of women in their 30s, 15.0% in their 40s, 32.6% in their 50s, and 50.2% in their 60s) to the extent that half of the women over the age of 60 had hypertension [2]. 

The first action for BP control is BP measurement. Because the results of BP measurement are influenced by multiple factors including time of day, location, and activity level, the accurate measurement and interpretation of BP are critical to diagnose and track hypertension. Accordingly, home BP measurements by the patient and 24-hour ambulatory BP measurements in addition to simple office sitting BP measurements are meaningful [3]. The measurement of 24-hour ambulatory BP in hypertensive patients aims to identify the degree of nocturnal decrease in BP, leading to the increased interest in quality of sleep, sleep disorders, and duration of sleep. In general, night-time BP decreases due to increased parasympathetic nervous system activity and decreased sympathetic nervous system activity during sleep [3]. Thus, the presence of sleep disorders, the duration of sleep, and the quality of sleep can influence hypertension [4]. 

The management of hypertension aims at preventing end organ complications and mortality due to hypertension, with the target BP below 140/90 mmHg [3, 5]. The goal of nursing intervention is to increase patient understanding of the hypertension process and its treatment and increase patient involvement in self-management programs to ultimately prevent hypertension complications [5]. 

Recently, there has been an increasing interest in holistic interventions combining traditional and complementary therapies for health promotion and maintenance. Aromatherapy is a form of complementary therapy using essential oil extracted from plants. The oil is applied through different means to achieve various effects through its chemical properties and application methods [6, 7]. Of the aromatherapy essential oils, lavender (*Lavandula officinalis*), marjoram (*Origanum majorana*), ylang-ylang (*Cananga odorata*), and Neroli (*Citrus aurantium*) are used for hypertensive patients. Lavender is the selective oil that balances the nervous system and alleviates insomnia, while marjoram activates parasympathetic nervous system and relaxes the sympathetic nervous system. Ylang-ylang controls cardiac palpitations and hypertension, and Neroli oil is effective in insomnia and depression. The main chemical ingredients of lavender, marjoram, ylang-ylang, and neroli are linalyl acetate, terpinen-4-ol, benzyl acetate, and limonene, respectively [6]. 

Aroma massage combines massage with essential oils to stimulate blood and lymphatic circulation and improve oxygen and nutrient supply, and it is effective for the relaxation of tense muscles, pain relief, and alleviation of emotional disturbances [6, 8, 9]. Women in middle age experience changes in the autonomic nervous system such as increases in adrenal cortex hormones and catecholamines, leading to increased BP and unsuccessful psychological management, which can have effects such as depression, anxiety, and insomnia [10, 11]. Recent research on the effects of aromatherapy on BP and sleep has targeted menopausal women [12], prehypertensive middle-aged women [13], and stage 1 hypertensive patients [14]. But there have been no studies using aroma massage and body cream as a method of aromatherapy. 

Thus, the aim of this study was to evaluate the effect of aroma massage on home BP, office BP, ambulatory BP, and sleep quality.

## 2. Methods

### 2.1. Study Design

**Figure 1:**
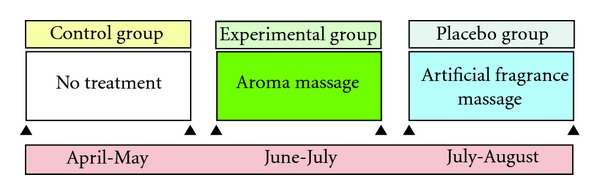
Study design.

This study is a nonequivalent control group, nonsynchronized study intended to compare the effects of aroma massage on home BP, office BP, ambulatory BP, and sleep in middle-aged women with hypertension (Figure 1). To prevent the spread and contamination of data as a part of the nonsynchronized study design, the control group data were collected before the data from the intervention group (aroma massage group) and the placebo group (artificial fragrance massage group).

### 2.2. Participants and Data Collection

The participants were recruited between March 15 and June 30 of 2011 at E University Hospital in D city, outpatient clinic in the department of internal medicine. Ethical approval was obtained from the institutional review board (IRB) after submission of the research plan. A notice was sent to the department, and the participants were recruited from patients diagnosed with hypertension and receiving ongoing outpatient cardiology followup after consultation with an internal medicine specialist. The eligibility criteria included the following: (1) a diagnosis of hypertension and ongoing followup treatment or consultation at outpatient cardiology clinic; (2) women between the ages of 40 and 59; (3) ability to communicate and describe symptoms; (4) not currently taking anxiolytics or hypnotics; (5) no presence of psychiatric disorders; and (6) providing informed consent regarding the experiment.

The participants were recruited from hypertensive patients using a notice sent to the Internal Medicine Outpatient Clinic; their gender, medications, and conformity to the eligibility criteria were reviewed. The study interventions, measurement methods, and the ability to enroll and drop out of the study were explained to participants, and consent was obtained from those in agreement. The participants were instructed to visit the research office on the intervention day according to the schedule and avoid excessive exercise and diet during the study period and to notify the researchers when there was a change in antihypertensive medication prescription. 

### 2.3. Sample Size Calculation

The sample size of this study was determined using the G* power program and by assigning alpha value, power, and effect size. The effect size was determined based on a prior aromatherapy study [14] and assigning mean, sample size, and pooled variance in the G* power program. To calculate the sample size, we substituted *α*(0.05), 1-*β* (0.80), group (3), and effect size (0.34). The sample size was determined to be a total of 90 participants in the three groups using G* power. In addition, estimating a dropout rate of 10%, 33 participants were assigned to the experimental group, the control group, and the placebo group, for a total of 99 participants. 

### 2.4. Interventions

The intervention used in the experimental group was the application of aroma massage and aroma body cream. The placebo group received massage and body cream with an artificial fragrance, and the control group did not receive any intervention. According to the schedule, there were a total of five sessions in the research office for four weeks. The aroma massage oil used contained essential oils prescribed by an international aromatherapist prepared by blending lavender, marjoram, ylang-ylang, and Neroli at a ratio of 20 : 10 : 15 : 2 followed by dilution to 3% with a carrier oil prepared by blending almond oil and jojoba oil at ratio of 9 : 1. Such prepared massage oil was preserved at room temperature for use. The aroma body cream was synthesized according to the prescription of an international aromatherapist. The oil base was prepared by combining 100 mL of jojoba oil, 100 mL of sweet almond oil, 100 mL of evening primrose oil, and 30 mL of olive wax. The water base was prepared by adding 700 mL of rose water and 20 g of vitamin E in a glass beaker and heating in boiling water until the temperature reached over 70~75°C, at which point the water base was added to the oil base. The water base combined with oil base was mixed using a blender for 10–15 minutes until the temperature reached below 40°C, at which point the blending oil used for massage (lavender, marjoram, ylang-ylang, and Neroli blended in 20 : 10 : 15 : 2 ratio) was added to produce a 3% diluted body cream. 

Four research assistants providing massage were taught aroma massage theory and massage protocols (4 hours) and received practical training (4 hours) from an international aromatherapist. The assistants performed practice massages before providing massages to participants according to the massage protocols. Aroma massage was provided in a research office that was divided into a waiting room and a massage room for study purposes at E University. According to the schedule, once the study participants arrived at the office, they changed into a massage gown and relaxed in a supine position on the bed for 10 minutes followed by BP measurement using a digital sphygmomanometer (Omron, HEM-780, Japan) as a preintervention measurement and massage according to the massage protocol. The massage was performed in the order of back, posterior legs, anterior legs, abdomen, arms, and shoulders. The total amount of oil needed for the massage was approximately 30 mL, and the duration of massage was approximately 1 hour. Ten minutes after the massage was completed, another BP measurement was taken as a postinterventional BP using the same method as the preinterventional measurement, and the participants were provided with 150 mL of lukewarm water to drink. A total of 200 mL of prepared aroma body cream was provided to the participants after the first massage, with instructions to apply approximately 10 mL on arm, legs, and abdomen, excluding the chest and back, after showering and before sleep every day.

Artificial fragrance massage oil and body cream were prepared and used for the placebo group. Almond oil and jojoba oil were blended in a 9 : 1 ratio to produce the carrier oil, in which artificial fragrance was diluted to 3%. The massage oil was stored at room temperature for use. The artificial fragrance body cream was produced and applied by the researchers and research assistants as the same method of making a massage oil and body cream. The control group received no intervention.

### 2.5. Outcome Measures

#### 2.5.1. Blood Pressure

Home BP was measured by participants every Tuesday and Friday at 10 AM after 10 minutes of rest using a home digital BP machine. Two measurements were performed on the left upper arm, and the average of the BP measurements was calculated. The 24-hour ambulatory BP was measured using a 24-hour ambulatory BP monitor (AND TM-2430, Japan). Daytime BP was measured every 30 minutes between 6 AM and 9:30 PM, while the nighttime BP was measured every hour between 10 PM and 6 AM the next morning. These measurements were performed once before and once after the intervention.

#### 2.5.2. Sleep Quality

To assess sleep quality in this study, a translated version of Verran and Synder-Halpern (VSH) Sleep Scale (1987) by Kang (1992) was used. The tool assesses four categories related to beginning of sleep and depth of sleep for a total of 8 questions each on a scale of 0–10 (a total range of 0–80), with higher score corresponding to a higher sleep satisfaction. 

### 2.6. Data Collection and Analyses

Prior to beginning the study, the participants were asked to relax for 10 minutes before measuring their preinterventional BP and obtaining sleep status and 24-hour ambulatory BP measurements. To test the effects of aromatherapy essential oil massage, the experimental group received massage once a week using diluted blended essential oil and daily application of body cream, while the placebo group received a massage with carrier oil with artificial fragrance once a week and daily application of body cream. The control group received no interventions. To measure the postinterventional effect, twice-weekly home BP was measured until week 3; in the fourth week, home BP, 24-hour ambulatory BP, and sleep status were evaluated.

The statistical analysis of the data was performed using SPSS 19.0 software. The general characteristics of participants were analyzed in terms of frequency, number, and percentage using *χ* ^2^-test and ANOVA. The test for homogeneity on preinterventional dependent variables between the experimental group, the placebo group, and the control group was conducted using ANOVA. The pre- and postinterventional BP and the sleep status of the participants in the experimental, placebo, and control groups were analyzed using ANOVA, repeated measures ANCOVA, and *χ* ^2^-test, while the post-hoc analysis was performed with the Tukey method. The reliability of the measurement tool for quality of sleep was analyzed using internal reliability Cronbach's *α* value.

## 3. Results

### 3.1. Participants

A total of 99 eligible participants were recruited to the study. Of them, three patients were lost to followup in the experimental group due to home situations, one patient was admitted with psychiatric issues, and one had a change in BP medication, leading to a final total of 28 participants in the experimental group. The placebo group had three patients lost to followup due to home situations, one due to antihypertensive medication changes, and one had mild pruritus and dropped out after two attempts for participation, leading to a final total of 28 participants. The control group had four participants lost to followup due to home situation, one patient who refused to participate due to inability to perform 24-hour ambulatory BP monitoring at work, and one patient who could not participate due to overseas travelling, leading to a final total of 27 participants. Therefore, there were 83 members in the study analyzed as final eligible study participants.

### 3.2. Homogeneity between the Groups

The general characteristics of the participants in the experimental, placebo, and control groups are shown in Table 1. No statistical differences were found between the groups with regards to marital status, level of education, presence of menopause, drinking, smoking, and exercise. There were also no significant differences in age, height, weight, age of menarche, age of first birth, baseline SBP, baseline DBP, and baseline pulse rate.

### 3.3. Effect of Aroma Massage on Home BP

The SBP and DBP were measured twice weekly for a total of eight measurements to evaluate the effect of aroma massage on participant home BP (Table 2). The SBP measurements over four weeks did not show significant differences with time, and there was no interaction between the groups and time on repeated measures ANCOVA using initial SBP as a covariate. However, there were significant differences in repeated measurements of SBP depending on the group (*F* = 6.71, *P* = 0.002). The post-hoc analysis with the Tukey method showed that the experimental group showed significant differences compared to the placebo group and the control group (*P* < 0.05).

The eight DBP measurements over four weeks did not show significant differences with time, and there was no interaction between the groups and time on repeated measures ANCOVA using initial DBP as a covariate. There was no difference in DBP measurements within the 3 groups.

### 3.4. Effect of Aroma Massage on Office BP

To determine the immediate effect of aroma massage, BP measurements were taken before the massage in the office followed by SBP measurement 10 minutes after the massage to compare with the office BP. The postintervention office SBP, measured after each of the five massages, did not show significant differences between the two groups after the first massage. The aroma massage group showed significantly reduced SBP after the second (*t* = −3.444, *P* = 0.001), third (*t* = −2.65, *P* = 0.01), fourth (*t* = −3.33, *P* = 0.002), and fifth sessions (*t* = −4.87, *P* < 0.001). There was statistical significance in groups × time (*F* = 3.63, *P* = 0.007) and the groups (*F* = 13.34, *P* = 0.001), while there was not statistically significant in time. 

Postintervention office DBP after the first session did not show significant differences between the two groups. However, there were significant differences between groups after the second (*t* = −3.09, *P* = 0.003), third (*t* = −2.08, *P* = 0.04), fourth (*t* = −2.38, *P* = 0.02), and fifth sessions (*t* = −3.76, *P* < 0.001) in office DBP. The results of repeated measures ANOVA showed significant difference with time (*F* = 3.84, *P* = 0.005), groups (*F* = 8.46, *P* = 0.005), and group × time (*F* = 2.79, *P* = 0.03) (Table 3).

### 3.5. Effect of Aroma Massage on 24-Hour Ambulatory BP

The 24-hour ambulatory BP was measured before and after the intervention for the experimental, placebo, and control groups. There were no significant differences between groups in 24-hour ambulatory daytime BP and 24-hour ambulatory nocturnal BP (Table 4). 

### 3.6. Effect of Aroma Massage on Sleep Quality

There were significant differences between groups (*F* = 6.75, *P* = 0.002) after intervention (Table 5). There were significant differences in changes of sleep quality between groups (*F* = 9.32, *P* < 0.001). In a post-hoc analysis, the aroma massage group showed significant improvement in sleep quality compared with placebo and no-treatment groups (Tukey, *P* < 0.05).

## 4. Discussion

This study investigated the effects of aroma massage on home BP, 24-hour ambulatory BP, and sleep quality in middle-aged women with hypertension. The results show that aroma massage significantly reduced home SBP compared with the placebo and no-treatment groups. 

The aroma massage group showed an approximate reduction of 15 mmHg for home SBP, while the placebo group showed an approximate reduction of 6 mmHg. One previous study showed a reduction of 4 mmHg in SBP using posterior neck massage three times a week for six weeks with lavender oil [15]. Comparing with these results, the effect observed with once-weekly overall body massage in this study shows that it is very effective in lowering BP. The oils used in this study (a blend of lavender, ylang-ylang, marjoram, and Neroli) are thought to have produced synergistic effects in lowering BP compared with a simple lavender oil [13]. In addition, previous research using inhalation therapy using lavender, ylang-ylang, and bergamot showed a decrease of 19/10 mmHg in BP and showed synergistic effects in lowering BP compared to this study [14].

The aroma massage may reduce BP by inducing the physical relaxation and decreasing activation of sympathetic nervous system. In this study, the placebo group with artificial fragrance also showed decreased BP with massage. This is thought to be due to the effect of massage rather than the artificial fragrance.

In particular, the office SBP decreased by 12 mmHg after the first massage and 11 mmHg after the fifth massage in the aroma massage group. This shows the acute effect of aroma massage for immediate decreasing of BP. The BP decrease after the first massage in the placebo group was approximately 7 mmHg. However, it did not show substantial reduction as massage sessions progressed, pointing to a temporary effect of massage with artificial fragrance. 

The home DBP decreased by approximately 4 mmHg in the experimental group, 3 mmHg in the placebo group, and 1 mmHg in the control group, showing no difference among the groups. The office DBP before and after massage showed a reduction of approximately 5 mmHg in the experimental group as opposed to an elevation of 2 mmHg in the placebo group. The effect of aroma massage in reducing DBP is limited, although there appears to be some immediate reducing effect of aroma massage for office DBP.

There was no difference in 24-hour ambulatory BP between groups. A once-weekly intervention of aroma massage and cream application does not appear to promote positive 24-hour ambulatory BP. Therefore, further research on the frequency of massage and the active ingredients and types of oils used is necessary.

In terms of sleep quality, the aroma massage group showed an improvement, while the placebo and no-treatment groups failed to do so. This result appears to be due to the effect of the essential oil used in this study on the autonomic nervous system to increase the quality of sleep [7, 9]. These results are in line with those of a study by Jung and Jeon [16]. They used a mixture of lavender, bergamot, and clary sage in a 3 : 2 : 1 ratio applied to hemiplegic patients on the arm, hand, and the lower neck through aroma massage and showed reduction of sleep disturbance score. The results of this study are consistent with the other study of middle-aged women using a lavender essential oil necklace and one to two drops of lavender essential oil on the pillow before sleep (instead of the necklace) [17], which showed improvement in sleep quality.

Our study did not take into account the preference of participants for aroma fragrance. Although most participants reported a good feeling toward the aroma, some reported preference for another fragrance. Thus, the psychological and physical responses may vary depending on the fragrance preference of the subject receiving the aromatherapy.

In conclusion, aroma massage was effective in reducing home SBP and immediate office BP before and after the intervention as well as increasing sleep quality in middle-aged women with hypertension. Further research comparing the effects of various and convenient aromatherapy methods including bathing, topical application, and foot washing is needed.

## Figures and Tables

**Table 1:** Homogeneity between groups.

Categories	Aroma (*n* = 28)	Placebo (*n* = 28)	Control (*n* = 27)	*χ* ^2^ or *F*	*P *
	Mean ± SD				

Age (yr.)	52.5 ± 4.6	54.1 ± 3.8	53.3 ± 4.6	1.01	.37
Height (cm)	157.1 ± 6.9	158.0 ± 4.9	158.4 ± 3.8	0.44	.68
Weight (kg)	61.8 ± 7.4	63.6 ± 7.6	64.2 ± 7.0	0.81	.45
Age of menarche (yr.)	15.6 ± 1.68	15.4 ± 1.9	15.6 ± 1.8	0.15	.86
Age of first birth (yr.)	26.1 ± 4.3	25.8 ± 3.6	26.6 ± 4.5	0.22	.80
First SBP (mmHg)	128.1 ± 12.2	127.5 ± 14.4	121.5 ± 13.1	2.06	.13
First DBP (mmHg)	79.7 ± 9.5	80.4 ± 11.3	80.1 ± 10.6	0.04	.96
First PR (bpm)	69.2 ± 8.7	71.0 ± 12.4	70.8 ± 8.6	0.26	.77

	*N* (%)				

Marital status					
Married	26 (92.9)	26 (92.9)	22 (81.5)	3.44	.30
Single	2 (7.1)	2 (7.1)	5 (18.5)		
Education					
Middle school	4 (13.3)	8 (28.6)	5 (18.5)	2.84	.58
High school	13 (46.4)	12 (42.9)	15 (55.6)		
University	11 (39.3)	8 (28.6)	7 (25.9)		
Presence of menopause					
Postmenopausal	14 (50.0)	15 (53.6)	17 (65.4)	3.79	.47
Intramenopausal	8 (28.6)	10 (35.7)	4 (15.4)		
No menopausal	6 (21.4)	3 (10.7)	5 (19.2)		
Drinking					
No	18 (64.3)	18 (64.3)	17 (63.0)	0.01	.99
Yes	10 (35.7)	10 (35.7)	10 (37.0)		
Smoking					
No	27 (96.4)	28 (100)	25 (92.6)	2.17	.34
Yes	1 (3.6)	0 (0.0)	2 (7.4)		
Exercise					
No exercise	5 (17.9)	8 (28.6)	7 (25.9)	2.13	.71
Less than two times per week	10 (35.7)	12 (42.9)	10 (37.0)		
More than three times per week	13 (46.4)	8 (28.6)	10 (37.0)		

DBP: diastolic blood pressure; PR: pulse rate; SBP: systolic blood pressure.

**Table 2:** Aroma massage on home BP.

Time	Aroma (*n* = 28)	Placebo (*n* = 28)	Control. (*n* = 27)	*F*	*P*
SBP					

Baseline	128.1 ± 12.2	127.5 ± 14.4	121.5 ± 13.1	2.06	0.13
1st week					
Tue.	120.7 ± 14.1	121.2 ± 12.4	125.7 ± 12.3	1.24	0.29
Fri.	118.5 ± 13.8	122.8 ± 13.7	123.3 ± 11.0	1.12	0.33
2nd week					
Tue.	120.6 ± 11.0	121.6 ± 12.3	122.4 ± 11.9	0.16	0.85
Fri.	117.9 ± 10.1	117.6 ± 13.4	121.7 ± 11.3	1.06	0.35
3rd week					
Tue.	116.0 ± 12.5	118.6 ± 12.7	122.3 ± 11.8	1.77	0.18
Fri.	114.7 ± 12.6	119.5 ± 11.1	121.9 ± 12.0	2.65	0.08
4th week					
Tue.	115.6 ± 9.3	121.6 ± 12.1	122.2 ± 11.1	3.21	0.05
Fri.	113.3 ± 11.0^a^	121.8 ± 11.3^b^	120.9 ± 12.1^b^	4.66	0.01

Group: *F* = 6.71, *P* = 0.002; time: *F* = 0.46, *P* = 0.86; group × time: *F* = 1.68, *P* = 0.07					

DBP					

Baseline	79.7 ± 9.5	80.4 ± 11.3	80.1 ± 10.6	0.04	0.96
1st week					
Tue.	79.0 ± 8.4	77.8 ± 9.1	81.5 ± 11.0	1.06	0.96
Fri.	78.8 ± 9.9	79.4 ± 10.1	81.5 ± 11.0	0.26	0.67
2nd week					
Tue.	80.9 ± 6.6	78.7 ± 6.6	79.2 ± 9.8	0.52	0.36
Fri.	79.9 ± 8.3	77.0 ± 10.2	79.6 ± 9.1	0.86	0.21
3rd week					
Tue.	79.4 ± 8.7	76.4 ± 11.5	81.0 ±10.4	1.45	0.13
Fri.	77.9 ± 9.5	76.8 ± 11.0	80.1 ± 7.6	0.91	0.40
4th week					
Tue.	77.5 ± 9.1	78.2 ± 10.1	79.4 ± 8.6	0.29	0.90
Fri.	75.9 ± 8.1	77.4 ± 10.5	79.4 ± 10.1	0.89	0.66

Group: *F* = 1.02, *P* = 0.37; time: *F* = 0.53, *P* = 0.81; group × time: *F* = 1.46, *P* = 0.13					

Values are expressed as mean ± standard deviation.

Means for each group with different superscript (a, or b) indicate a significant difference (Tukey test; *P* < 0.05).

BP: blood pressure; DBP: diastolic blood pressure; PR: pulse rate; SBP: systolic blood pressure.

**Table 3:** Aroma massage on office BP.

Time	Aroma (*n* = 28)	Placebo (*n* = 28)	*t*	*P*

SBP				

1st session				
Pre	124.0 ± 11.1	125.8 ± 15.7	−0.49	0.63
Post	112.8 ± 12.4	118.9 ± 15.2	−1.63	0.11
2nd session				
Pre	119.0 ± 11.7	121.4 ± 11.5	−0.78	0.44
Post	108.5 ± 10.8	119.7 ± 13.3	−3.44	0.001
3rd session				
Pre	118.2 ± 10.7	122.3 ± 13.1	−1.28	0.21
Post	109.7 ± 11.1	118.6 ± 13.9	−2.65	0.01
4th session				
Pre	119.0 ± 10.3	125.1 ± 15.5	−1.75	0.09
Post	111.1 ± 9.9	122.9 ± 15.9	−3.33	0.002
5th session				
Pre	119.4 ± 12.1	126.6 ± 15.7	−1.93	0.06
Post	108.9 ± 9.7	125.0 ± 14.6	−4.87	<0.001

Group: *F* = 13.34, *P* = 0.001; time: *F* = 2.15, *P* = 0.08;				
group × time: *F* = 3.63, *P* = 0.007				

DBP				

1st session				
Pre	74.3 ± 7.7	76.0 ± 10.9	−0.67	0.51
Post	71.8 ± 8.4	74.7 ± 9.7	−1.19	0.24
2nd session				
Pre	72.1 ± 8.4	74.3 ± 8.0	−1.01	0.32
Post	67.8 ± 7.8	74.8 ± 9.3	−3.09	0.003
3rd session				
Pre	72.7 ± 7.7	73.7 ± 8.0	−0.50	0.62
Post	70.2 ± 7.8	74.6 ± 8.3	−2.08	0.04
4th session				
Pre	73.1 ± 6.8	76.0 ± 9.8	−1.27	0.21
Post	72.0 ± 6.7	77.4 ± 10.1	−2.38	0.02
5th session				
Pre	72.6 ± 8.3	76.0 ± 9.9	−1.38	0.18
Post	69.5 ± 6.4	78.1 ± 10.3	−3.76	<0.001

Group: *F* = 8.46, *P* = 0.005; time: *F* = 3.84, *P* = 0.005;				
group × time: *F* = 2.79, *P* = 0.03				

Values are expressed as mean ± standard deviation.

BP: blood pressure; DBP: diastolic blood pressure; SBP: systolic blood pressure.

**Table 4:** Aroma massage on 24-hour ambulatory BP.

Time	Aroma	Placebo	Control	*F*	*P*
	(*n* = 28)	(*n* = 28)	(*n* = 27)		
SBP					

Daytime					
Pre	134.8 ± 12.0	136.2 ± 13.5	128.5 ± 12.5	2.88	.06
Post	130.3 ± 11.7	128.4 ± 12.2	133.9 ± 12.9	1.37	.26
Night time					
Pre	122.2 ± 15.8	124.9 ± 13.9	119.1 ± 16.4	0.98	.38
Post	117.5 ± 15.6	119.0 ± 9.9	122.9 ± 18.1	0.93	.40

DBP					

Daytime					
Pre	82.7 ± 8.6	83.6 ± 8.1	81.6 ± 9.4	0.37	.69
Post	81.4 ± 8.3	80.0 ± 8.1	83.4 ± 8.9	1.07	.35
Night time					
Pre	74.8 ± 9.9	76.4 ± 8.9	74.4 ± 11.1	0.30	.74
Post	73.6 ± 10.5	71.7 ± 6.8	74.6 ± 11.0	0.59	.55

Values are expressed as mean ± standard deviation.

BP: blood pressure; DBP: diastolic blood pressure; SBP: systolic blood pressure.

**Table 5:** Aroma massage on sleep quality.

Time	Aroma	Placebo	Control.	*F*	*P*
	(*n* = 28)	(*n* = 28)	(*n* = 27)		
Pre	51.0 ± 12.6	48.1 ± 15.8	55.6 ± 10.5	2.24	0.11
Post	58.1 ± 11.3^a^	46.5 ± 13.8^b^	50.3 ± 10.9^b^	6.75	0.002
Difference	7.1 ± 12.6^a^	−1.6 ± 9.6^b^	−5.3 ± 10.6^b^	9.32	<0.001

Values are expressed as mean ± standard deviation.

Means for each group with different superscript (a, b, or c) indicate a significant difference (Tukey test; *P* < 0.05).
